# Oral intake recovery following salvage laryngectomy with circumferential free flap reconstruction in a patient with prior pharyngogastric anastomosis and chemoradiation

**DOI:** 10.1093/jscr/rjag852

**Published:** 2026-09-29

**Authors:** Chloe E Coulter, Samuel J Hopper, Gina D Jefferson

**Affiliations:** University of Mississippi School of Medicine, 2500 N State Street, Jackson, MS 39216, United States; Department of Otolaryngology–Head and Neck Surgery, University of Mississippi Medical Center, 2500 N State Street, 5 East, Jackson, MS 39216, United States; Department of Otolaryngology–Head and Neck Surgery, University of Mississippi Medical Center, 2500 N State Street, 5 East, Jackson, MS 39216, United States

**Keywords:** esophageal squamous cell carcinoma, laryngectomy, laryngopharyngectomy, pharyngogastric anastomosis, microvascular free tissue flaps, chemoradiotherapy, dysphagia

## Abstract

Salvage laryngopharyngectomy in patients with a prior history of chemoradiation and surgery constitutes a substantial reconstructive challenge. Functional recovery is often limited by compromised vascular integrity combined with loss of pharyngeal mucosa, contributing to high rates of post-operative complications. We present a patient with squamous cell carcinoma of the esophagus, previously managed with chemoradiation and salvage esophagectomy with pharyngogastric anastomosis. She developed a second recurrence involving the pyriform sinus adjacent to the neoesophagus, requiring salvage laryngopharyngectomy and circumferential free flap reconstruction. An anterolateral thigh microvascular free flap was anastomosed to the gastric conduit. Despite significant risk factors of anatomic defect and hypovascularity, the patient successfully regained oral intake. This case demonstrates that favorable functional outcomes are possible in medically complex patients undergoing salvage reconstruction.

## Introduction

Salvage laryngopharyngectomy carries significant morbidity and presents unique reconstructive challenges, particularly in the setting of prior chemoradiation to the head and neck or prior surgical treatments [1, 2]. With circumferential pharyngeal defects, anatomical distortion limits surgical options and complicates swallowing restoration. Radiation induces fibrosis and impairs wound healing, while chemotherapy further increases postoperative complication risk through immunosuppression and decreased synthesis of blood vessels and extracellular matrix [3]. Free flap reconstruction, one of the most technically demanding procedures, is the principal means of restoring alimentary continuity for circumferential defects [4]. Reconstruction is further complicated by altered anatomy, such as a gastric conduit following esophagectomy. We present a patient with advanced mid-esophageal squamous cell carcinoma (SCC) previously treated with chemoradiation and eventual salvage esophagectomy with pharyngogastric anastomosis. She later developed locoregional recurrence at the pyriform sinus involving the neoesophagus, necessitating salvage laryngopharyngectomy and circumferential free flap reconstruction. The patient now enjoys successful restoration of oral intake and remains disease-free.

## Case report

A 54-year-old woman with heavy alcohol use and a 10 pack-year smoking history was diagnosed with cT3N0M0 mid-esophageal SCC in 2017. She achieved a 7-year remission after initial concurrent chemoradiation. In 2023, dysphagia and weight loss led to diagnosis of recurrent disease, treated with neoadjuvant chemotherapy and immunotherapy followed by salvage esophagectomy with pharyngogastric anastomosis in May 2024. Adjuvant chemotherapy and immunotherapy followed salvage surgery. During systemic therapy in February 2025, interval computed tomography (CT) chest/abdomen/pelvis demonstrated a right kidney lesion that enlarged 3 months later. She underwent right nephrectomy in June 2025 for this single oligometastatic disease, followed by additional systemic therapy.

PET/CT 3 months later showed concerning hypopharyngeal uptake (Fig. 1). Direct laryngoscopy showed a mass filling the right pyriform sinus extending to the posterior pharyngeal wall and neoesophageal inlet. Biopsy was consistent with SCC. Multidisciplinary tumor board recommended salvage laryngopharyngectomy with anterolateral free flap reconstruction.

**Figure 1:**
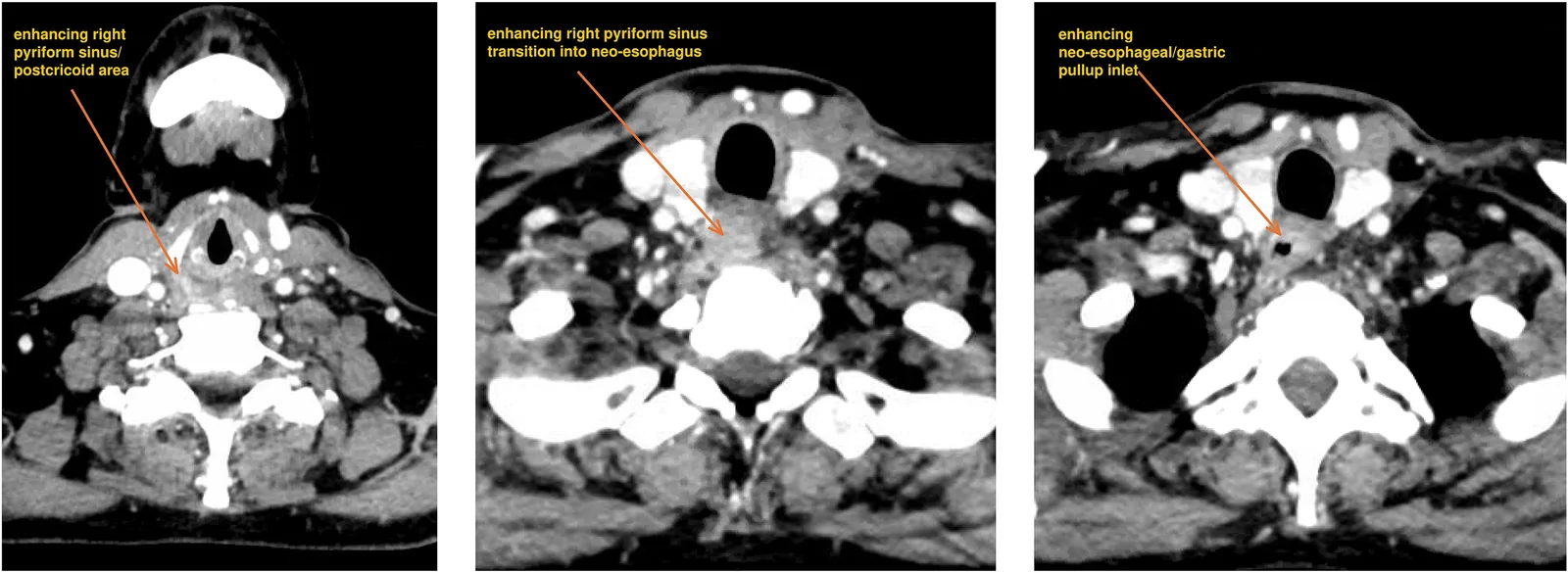
Preoperative contrast-enhanced CT scan of the neck. Series of CT images demonstrate contrast-enhancing, biopsy-proven squamous cell carcinoma at the right pyriform sinus, post-cricoid region extending into the neoesophageal gastric pull-up.

She underwent salvage laryngopharyngectomy, cervical esophagectomy, and bilateral selective neck dissections (levels II–IV and VI). Final pathology revealed moderately differentiated invasive SCC of the cervical neo-esophagus, extending into the right pyriform sinus. The tumor measured 3.3 cm with focal perineural invasion. Margins were negative, with tumor within 1 mm of the neo-esophageal soft tissue margin. Nodal evaluation showed low-volume metastatic disease. Only one positive lymph node was found in each of the left level III (1/6) and right level IIa (1/8) dissections, without extranodal extension. Pathologic staging was ypT3N1. Reconstruction was performed using an anterolateral thigh free flap to repair the circumferential pharyngeal defect and reestablish continuity with the gastric conduit (Fig. 2).

**Figure 2:**
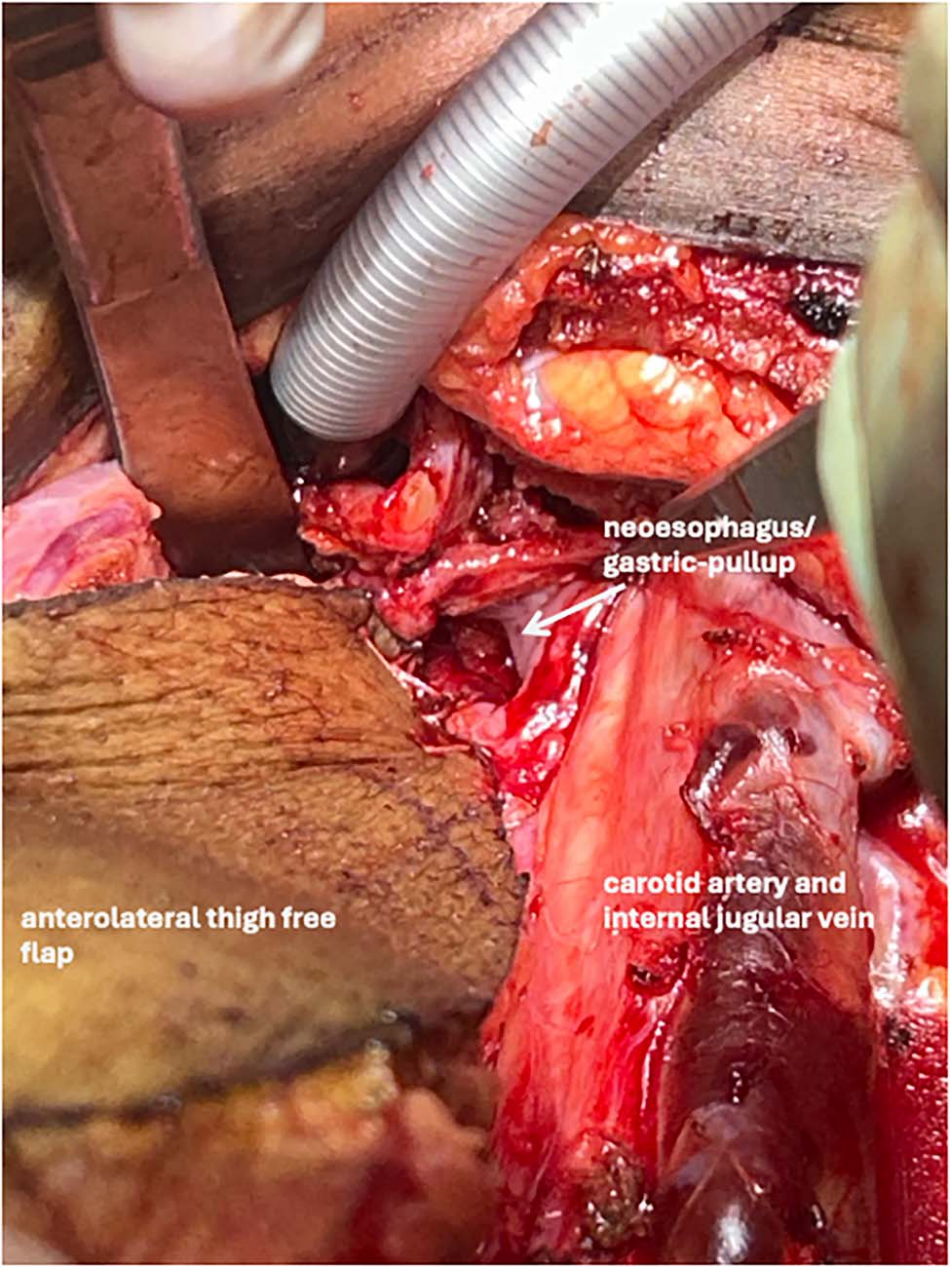
Intraoperative inset initiation of anterolateral thigh flap into the remaining neoesophagus/gastric pullup.

Her postoperative course was complicated by a pharyngocutaneous fistula managed conservatively for 3 weeks. An esophagram following adequate wound care demonstrated no leak, stricture, or obstruction, and a liquid oral diet was initiated. The patient currently tolerates oral intake with Functional Oral Intake Scale of 6 documented 4 months postoperatively. She completed adjuvant chemoradiation and is currently receiving maintenance chemotherapy and immunotherapy and currently has no evidence of disease 12 months postoperatively.

## Discussion

Head and neck cancer extirpation inherently presents challenges to restoration of form and function. Radiation exposure further exacerbates recovery secondary to ensuing fibrosis. After chemoradiation, salvage laryngopharyngectomy carries higher complication risk. Pharyngocutaneous fistula is the most common, around 30% [1, 3]. Introduction of non-radiated tissue for creation of the neopharynx aims to reduce fistula and wound problems while also re-establishing the swallowing conduit [3]. However, fasciocutaneous flaps lack a mucosal lining and any potential of peristalsis, resulting in delay in bolus propulsion. There is also an increased risk for stricture formation, particularly in previously radiated fields [5].

When multiple risk factors coexist in patients undergoing salvage laryngopharyngectomy, functional outcomes worsen. Several factors worsened our patient’s morbidity: prior chemoradiation, prior esophagectomy, the anatomical changes from a gastric pull-up, local recurrence involving the neo-esophagus, and postoperative fistula formation. The resultant circumferential defect required tubed free flap reconstruction. Healing was supported using a salivary bypass tube to stent the circumferential flap closure and to minimize occurrence of strictures. The defect was in an area with poor vasculature containing fibrotic, inelastic tissue, increasing tension and stricture risk. Circumferential defects in previously irradiated fields combined with a vulnerable pharyngo-gastric anastomosis characterized by tissue mismatch, impaired tissue compliance, and suboptimal perfusion predispose to anastomotic failure and extraluminal leak [6].

Complex reconstruction in this setting demands vascularized tissue. This anticipated defect drove the reconstruction choice, highlighting the limitations of a gastric pull-up, which were considered when exploring options to restore pharyngeal continuity. Currently, no single flap type independently predicts superior postoperative functional outcomes. Surgeons proficient in jejunal free flap transfer tout advantages of ability to reconstruct large defects and fast recovery of physiologic alimentation [7]. At our institution, the available microvascular surgeons do not perform jejunal free tissue transfer, leaving fasciocutaneous free flap options. A radial forearm free flap that is less bulky ideally would permit water-tight, circumferential reconstruction. However, the patient’s short stature with limited forearm circumference led to utilization of the bulkier anterolateral thigh free flap.

Reconstruction with free tissue transfer is associated with reduced rates of pharyngocutaneous fistula and improved functional recovery following laryngopharyngectomy in irradiated patients [8]. There is improved integrity of the local anastomotic junction through vascularized tissue and reduced mechanical stress, supporting more durable healing. Fistula formation does remain a potential complication which our patient briefly experienced. Both pharyngocutaneous fistulas and circumferential pharyngeal reconstruction increase risk for strictures and significantly impact swallowing recovery [9–11]. Swallowing outcomes vary, but our patient achieved 100% oral intake consisting of soft foods and shredded meats by 4 months postoperatively, has maintained her usual body weight 12 months following surgery, and has no evidence of disease.

Restoration of oral intake represents a meaningful functional endpoint in salvage laryngectomy and laryngopharyngectomy, reflecting recovery of swallowing function and overall postoperative outcome [8]. Despite compromised vascular and anatomical integrity, this patient regained oral intake following salvage laryngopharyngectomy with fasciocutaneous free flap reconstruction. This case illustrates that positive functional outcomes are attainable in medically complex patients with extensive prior treatment, particularly in the highly motivated patient with limited other medical co-morbidities.
